# Acute portal hypertension and oesophageal varices in pregnancy

**DOI:** 10.1177/1753495X261468841

**Published:** 2026-07-27

**Authors:** Sami Rayanne Lewis, Lisa Long, Karan Sampat

**Affiliations:** 1 Guy's, King's and St Thomas’ School Of Medical Education, 405987 King's College London, London, UK; 2 King's College Hospital NHS Foundation Trust, 8948 King's College Hospital, London, UK; 3 Dartford and Gravesham NHS Trust, 156489 Darent Valley Hospital, Kent, UK

**Keywords:** At least, portal hypertension, oesophageal varices

## Abstract

Non-cirrhotic portal hypertension (PHTN) is often attributed to portal vein thrombosis and may cause serious complications. Described here is a woman with a new portal vein thrombosis in her third pregnancy, complicated by PHTN and oesophageal varices. She was managed using a multi-centre, multidisciplinary approach throughout her pregnancy and delivered a healthy baby at term via induced vaginal birth. Here, the treatment modalities available for pregnant women with PHTN and the management of complications are discussed.

## Introduction

Portal hypertension (PHTN) may arise as a result of a variety of pre-, intra- and post-hepatic aetiologies.^1^ The main pre-hepatic cause is portal vein thrombosis, where a thrombus partially or completely obstructs the portal vein, leading to the hepatic venous pressure exceeding 5mmHg.^1,2^ This resistance to flow and lack of valves within the portal circulation results in decompression efforts from portacaval collaterals, such as the gastro-oesophageal veins, which become tortuous and dilated due to elevated venous pressure.^1^ Oesophageal varices develop, which are vulnerable to rupture, causing upper gastrointestinal haemorrhage.^3^ Fear of this bleeding risk renders a pregnancy with this phenomenon as high-risk, requiring specialist, multidisciplinary care in order to achieve the best clinical outcomes for mother and baby.^4^ In pregnancy, PHTN also carries an increased risk of miscarriage, premature birth as well as other obstetric complications such as HELLP syndrome.^5^

## Case description

A 32-year-old Caucasian woman was pregnant for the third time. She had a background of generalised anxiety disorder and previous deep vein thrombosis, assumed to be unprovoked with no evidence of predisposing factors. Her first pregnancy was complicated by pre-eclampsia and her second, by post-partum haemorrhage (PPH); both were spontaneous vaginal deliveries of live babies of healthy weight. Regular medications included lansoprazole, co-codamol and morphine; the indications for the latter two were unclear. She had previously been on citalopram which she stopped prior to pregnancy. She was not aware of ever having thrombophilia screening or pre-conception counselling. At booking, she was also taking co-amoxiclav for suspected cholecystitis.

This woman initially presented with abdominal pain, localised to the right upper quadrant and epigastric region, radiating across the abdomen and associated with rigors. She had lost over 5 kg over 10 days, but was not febrile or experiencing vomiting, diarrhoea, haematemesis or melaena; no stigmata of cirrhotic disease were noted. She was admitted for intravenous antibiotics for suspected cholecystitis and discharged soon after with oral antibiotics; at this time, her C-reactive protein (CRP) = 213 mg/L (normal range in pregnancy <10.0 mg/L). No abdominal imaging was performed at this stage. She was approximately 6 weeks of gestation at the time of this admission, but was not aware of the pregnancy until a later early pregnancy scan.

She re-presented to a different hospital at 9 + 6 weeks as her symptoms had worsened, despite treatment. Her CRP was persistently elevated, now 216.5 mg/L, rising to 238.3 mg/L soon after; her liver function tests were all normal. She was readmitted, and an abdominal ultrasound scan (USS) was performed, revealing complete portal vein occlusion by a thrombus, potential varices at the porta hepatis, fatty liver and free fluid in the abdomen; no portal pressures were documented. USS confirmed a viable intrauterine pregnancy. She was started on 10,000 units of dalteparin, twice daily, for a minimum of 3 months. Mild vaginal bleeding occurred following the initiation of treatment; a repeat pregnancy USS was reassuring. Owing to 's stability, intact hepatic function and absence of massive gastrointestinal bleed, thrombolysis and stenting of the portal vein were deemed unnecessary, in favour of regular surveillance through oesophagogastroduoudenoscopy (OGD) for varices. A repeat abdominal USS for resolution was also advised; imaging for abdominal wall varices was deemed unnecessary. This repeat USS demonstrated potential recanalisation of the portal vein and confirmed that the previously seen porta hepatis varices were in fact compensatory collateral vessels in response to the acute thrombotic occlusion. The woman was discharged after a week's admission.

**Figure 1.**
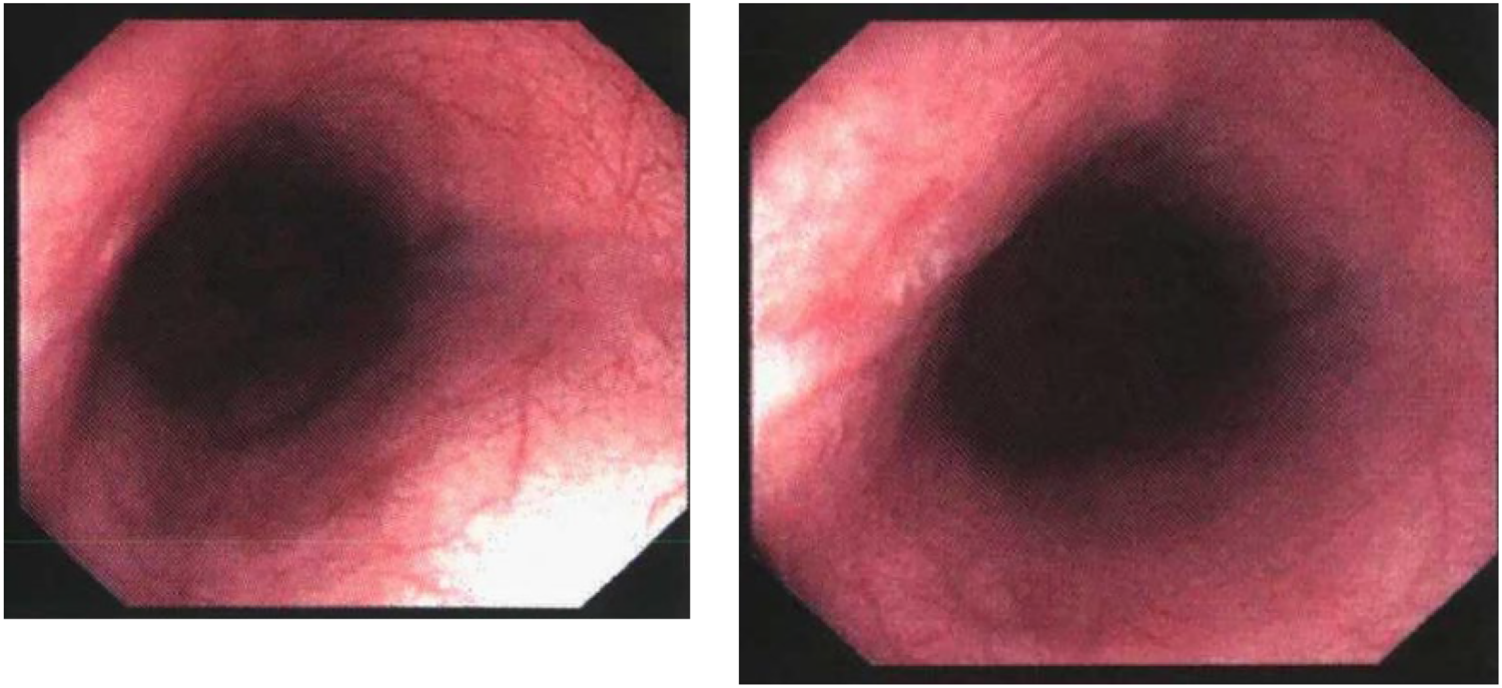
OGD images, superior to the GOJ where oesophageal varices were visualised; these disappeared on complete insufflation. OGD: oesophagogastroduoudenoscopy; GOJ: gastro-oesophageal junction.

A further USS, 10 days post-discharge revealed re-occlusion of the portal vein, resulting in re-admission to hospital at 12 + 2 weeks of gestation. An OGD exhibited two columns of barely visible varices at the gastro-oesophageal junction, disappearing on complete insufflation (Figure 1). Her dalteparin dose was amended, guided by anti-Xa level (target level 1.0–1.2 IU/mL). Propranolol (40 mg once daily) was added for variceal prophylaxis, and she was discharged. Her platelet count remained normal throughout pregnancy.

The OGD at 20 weeks of gestation was performed in the left lateral position and did not go beyond the stomach to minimise stress and examination time. This revealed minimal oesophageal varices and no fundal varices.

In view of her therapeutic anticoagulation, the induction of labour at 38 weeks of gestation was planned. The dalteparin was stopped 24 h prior to mitigate the risk of intra/PPH and allow for a safe window for neuraxial anaesthesia. A controlled vaginal dinoprostone release pessary (Propess) was inserted for 24 h, followed by two doses of Prostin (1 mg). No neuraxial anaesthetic was used. An intrapartum oxytocin infusion was started. The woman delivered a healthy baby boy weighing 3.5 kg, but sustained a second-degree perineal tear. An oxytocin infusion was started for 4 h, owing to her history of PPH, and rectal diclofenac was given for pain relief. Blood loss was minimal (estimated to be 300 ml), and she received 5000 units of dalteparin 6 h post-delivery. The following day, she was commenced on 10 mg of rivaroxaban once daily for life, and her propranolol was continued; she was presumed to have not been planning on breastfeeding.

## Discussion

It is essential to effectively manage oesophageal varices in pregnant women with PHTN, due to the catastrophic consequences associated with their rupture. The European Association for the Study of the Liver (EASL) strongly recommends regimented antenatal variceal surveillance as well as medical therapy to ease the PHTN and thus mitigate the risk of haemorrhage.^6^ Upper gastrointestinal endoscopy is considered to be the gold standard for monitoring and is safe to use in pregnancy – the small risk of fetal hypoxia can be alleviated by scoping in the left lateral position to prevent aortocaval compression.^6,7^ Endoscopic variceal ligation is used to treat actively bleeding vessels.^7^ Crocker et al. reported the case of a woman in her fifth pregnancy, who did not undergo early endoscopy. This woman experienced massive variceal bleeds, which required later OGDs with banding. This highlights the vital role that early surveillance plays in informing decision-making surrounding intervention, thus reducing the risk of complications.^8^

Non-selective beta-blocker therapy should also be initiated with the aim of decreasing portal pressure, thus slowing the development and reducing the risk of variceal rupture.^6,9^ In the pregnant woman, propranolol or carvedilol is recommended and is estimated to halve the risk of variceal haemorrhage.^6,10^ It should be noted that beta-blocker use in pregnancy has been shown to be associated with a small reduction in birthweight, as well as neonatal bradycardia and hypoglycaemia.^11^

Differences in opinion exist about the preferred mode of delivery in pregnant women with oesophageal varices, especially surrounding the safety of vaginal delivery. On one hand, the repetitive increases in intra-abdominal pressure, may aggravate the PHTN (thus increasing the risk of variceal bleeding) and therefore warrant caesarean delivery.^12^ Whilst this is not supported by guidelines or any randomised controlled trials, fear of peripartum oesophageal haemorrhage and resultant hepatic decompensation often pushes management towards this; a small 2014 case series reported that all (*n* = 7) pregnant women with oesophageal varices, regardless of obstetric condition, delivered via Caesarean section at term.^4,12^ However, the EASL guidelines state that vaginal delivery is preferable; however, an expedited second stage of labour (during which the risk pressure rises leading to variceal rupture is greatest) is recommended, and that a caesarean section should be performed for obstetric indications only.^6,7^ It is thought that in order to avoid excessive straining, which may precipitate variceal bleeding, extradural anaesthesia may be utilised in conjunction with instrumental techniques to accelerate the second stage of labour.^13^ In our case, variceal insignificance and the absence of any other obstetric considerations clearly supported vaginal delivery.

Portal vein thrombosis is generally uncommon in pregnancy.^8^ Previously reported cases highlight the high-risk nature of these pregnancies, especially complicated by portal vein thrombosis, as well as the role that optimal anticoagulation plays in combating thrombus development/recurrence and preventing consumptive coagulopathies, which may further complicate pregnancy and labour.^14,15^ This consideration is particularly relevant in this case; whilst the platelet count remained acceptable throughout, it was still closely monitored due to it being a key consideration in planning delivery and cessation of anticoagulation prior to this.

This case reinforces the pivotal role that multidisciplinary care has in improving her outcomes. Obstetrics (particularly maternal medicine), gastroenterology, haematology and anaesthetics are stalwart contributors in the management of pregnant women with PHTN, and effective communication is key for effective treatment to take place.^8,16^ Moreover, cross-institutional collaboration in the form of maternal medicine networks is also vital to ensure that appropriate levels of expertise are being applied to optimise her care.

## Conclusion

Portal vein thrombosis can cause PHTN, which can lead to the development of oesophageal varices which may rupture and cause catastrophic consequences. In the context of pregnancy, medical and surgical management, as well as mode of delivery must be meticulously planned and optimised by a multidisciplinary and collaborative approach in order to achieve the best outcomes.
